# Aspirin might reduce the incidence of pancreatic cancer: A meta-analysis of observational studies

**DOI:** 10.1038/srep15460

**Published:** 2015-10-21

**Authors:** Yan-Peng Zhang, You-Dong Wan, Yu-Ling Sun, Jian Li, Rong-Tao Zhu

**Affiliations:** 1 Institute of Hepatobiliary and Pancreatic Diseases, Zhengzhou University, Department of Hepatobiliary and Pancreatic Surgery, The First Affiliated Hospital of Zhengzhou University, School of Medicine, 1 Jianshe Road, Zhengzhou 450052, P.R. China; 2 Department of Integrated Intensive Care Unit, the First Affiliated Hospital of Zhengzhou University, School of Medicine, 1 Jianshe Road, Zhengzhou 450052, P.R. China

## Abstract

Although there is evidence that non-steroidal anti-inflammatory drugs (NSAIDs) might be able to prevent pancreatic cancer, the findings from epidemiological studies have been inconsistent. In this paper, we conducted a meta-analysis of observational studies to examine this possibility. We searched PubMed and Embase for observational (cohort or case-control) studies examining the consumption of aspirin and other NSAIDs and the incidence of or mortality rates associated with pancreatic cancer. Twelve studies including approximately 258,000 participants in total were analysed. The administration of aspirin significantly reduced the incidence of pancreatic cancer (8 studies; odds ratio (OR) = 0.77; 95% confidence interval (CI) = 0.62 to 0.96; *I*^2^ = 74.2%) but not the mortality associated with it (2 studies; OR = 0.94; 95% CI = 0.73 to 1.22). Specifically, frequent aspirin use was associated with reduced pancreatic cancer incidence (OR = 0.57; 95% CI = 0.39 to 0.83 for high frequency; OR = 0.57; 95% CI = 0.38 to 0.84 for medium frequency). The summary ORs regarding the incidence of pancreatic cancer and either non-aspirin NSAIDs use (OR = 1.08; 95% CI = 0.90 to 1.31) or overall NSAIDs use (OR = 0.97; 95% CI = 0.86 to 1.10) were not significant. In conclusion, aspirin use might reduce the incidence of pancreatic cancer; however, this finding should be interpreted with caution because of study heterogeneity.

Pancreatic cancer is the fourth leading cause of cancer-related death and one of the ten most common types of malignancies in the world^1^. In the United States, approximately 45,000 new cases of pancreatic cancer are diagnosed and 37,000 deaths occur each year; the survival rate is less than 1% 5 years after diagnosis^1^. Only approximately 10% of patients with pancreatic cancer are eligible for surgical resection, and the results of medical therapies remain unsatisfactory^2^. Therefore, an urgent need exists for a better understanding of the factors that are related to pancreatic cancer development and prognosis. Moreover, the identification of potential chemopreventive strategies for pancreatic cancer is highly desirable.

Cyclooxygenase-2 (COX-2) might play a role in cancer development^3^,^4^. Its expression is elevated in pancreatic carcinoma tissue compared with healthy pancreatic tissue^5^,^6^. Therefore, non-steroidal anti-inflammatory drugs (NSAIDs), which inhibit the COX-2 pathway, might hold promise for the chemoprevention and treatment of pancreatic cancer. In addition, use of NSAIDs (particularly aspirin) reduces the risk of several cancers, including colorectal^7^, breast^8^, gastric and esophageal^9^,^10^, lung^11^, and prostate cancers^12^. However, the findings from observational epidemiological studies regarding the relationship between aspirin/NSAIDs use and pancreatic cancer risk have been inconsistent^13^,^14^,^15^,^16^,^17^,^18^,^19^,^20^,^21^,^22^,^23^,^24^,^25^,^26^,^27^,^28^. Three meta-analyses have been published on this issue, each with different results. In 2006, Larsson *et al*.^29^ carried out a meta-analysis of 11 studies (one randomized controlled trial (RCT), three case-controls, and seven cohorts) and did not find associations between the risk of pancreatic cancer and aspirin, non-aspirin NSAIDs, or NSAIDs use. However, this evidence is not conclusive because the incidence and mortality of cancer were pooled to represent the overall risk of cancer. This strategy might obscure true decreases or increases in the incidence or mortality risk of pancreatic cancer associated with aspirin/NSAIDs use. Another meta-analysis^30^ that included 8 studies (three case-controls, four cohorts, and one RCT) did not show any association between aspirin/NSAIDs use and pancreatic cancer risk in low, intermediate, or high exposure groups. However, this evidence is limited because the aspirin and NSAIDs groups were pooled for the analysis, which might have masked the individual effects of aspirin versus other NSAIDs on the incidence of pancreatic cancer. More recently, Cui *et al*.^31^ conducted a meta-analysis that included 10 studies (four case-controls, five cohorts, and one RCT) and concluded that high-dose aspirin use reduces pancreatic cancer risk. However, this conclusion was not supported because the odds ratio (OR) of the high-dose aspirin group was not significant (odds ratio (OR) = 0.88; 95% confidence intervals (CI) = 0.76 to 1.01; *P* = 0.069)^31^.

Thus, these previous meta-analyses must be updated to revise the evidence concerning this issue. Moreover, the frequency and duration risks of aspirin use in real-world practice have not been systematically evaluated. Therefore, based on recent evidence, we performed a systematic review and meta-analysis of 12 observational studies to explore the possibility that NSAIDs use reduces the incidence of pancreatic cancer in real-world settings.

## Results

### Study selection and characteristics

**Figure 1:**
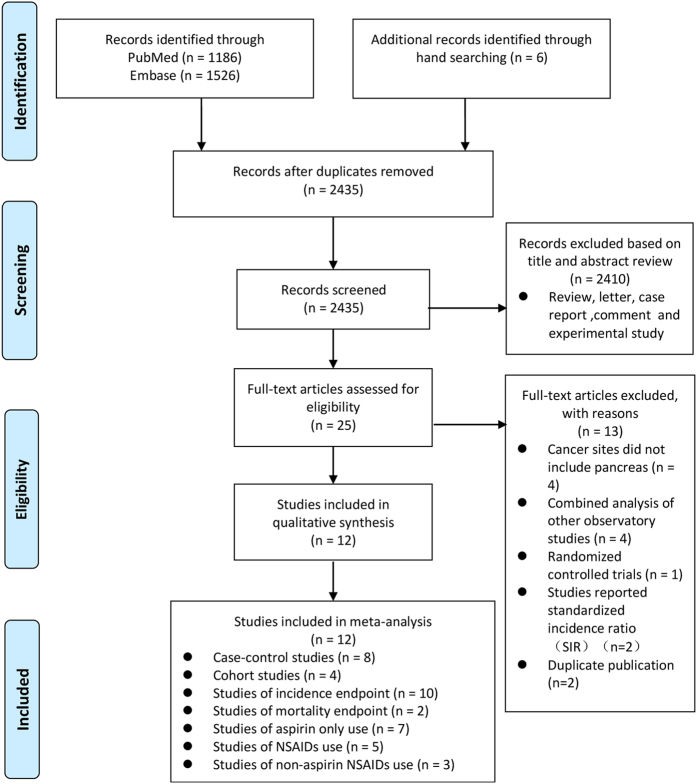
Article selection flow chart.

Our initial search yielded 2,718 potentially relevant publications, of which 283 duplicates were excluded. We then excluded 2,410 studies that were deemed irrelevant to the meta-analysis based on their titles and abstracts. After reviewing the full texts of the remaining 25 studies, we identified 12 studies for inclusion in the meta-analysis (for details see Fig. 1)^14^,^16^,^18^,^19^,^20^,^21^,^22^,^23^,^24^,^25^,^26^,^27^. Among reasons for excluding the 13 studies, specifically, we did not include Sørensen *et al*.^28^ or Friis *et al*.^13^ because these studies reported the standard incidence ratio (SIR) as effect size without adjustment. In addition, we excluded Schreinemachers *et al*.^15^ because its cohort was also included in Ratnasinghe *et al*.^16^. Moreover, Jacobs and colleagues conducted two cohort studies^17^,^23^ based on the same sample to investigate the effects of aspirin use on pancreatic cancer mortality; therefore, we excluded the study that included fewer cases and a briefer follow-up period^17^.

The main characteristics of the 12 included studies (8 case-control^18^,^19^,^20^,^22^,^24^,^25^,^26^,^27^ and 4 cohort studies^14^,^16^,^21^,^23^) are shown in Table 1. Approximately 258,000 participants were included in the current meta-analysis. Five studies (4 case-controls^20^,^24^,^25^,^26^ and 1 cohort^21^) evaluated overall NSAIDs use, 8 studies (6 case-controls^18^,^19^,^20^,^22^,^25^,^27^ and 2 cohorts^14^,^21^) addressed aspirin use, and 3 studies (1 case-control^19^ and 2 cohorts^14^,^21^) measured non-aspirin NSAIDs use with regard to the incidence of pancreatic cancer. Two cohort studies^16^,^23^ investigated the association between aspirin use and cancer-specific mortality. The years of publication of the included studies ranged from 2000 to 2014. Of the 12 included studies, 8 were conducted in the United States^14^,^16^,^19^,^21^,^22^,^23^,^26^,^27^, and 4 were completed in Europe^18^,^20^,^24^,^25^. In each study, the participants were either matched or the methodology was adjusted for a wide range of potential confounds. The average Newcastle-Ottawa Scales (NOS) quality score of the observational studies was 7.2 (range = 6 to 9). Greater detail is provided in Supplementary Table S1.

### Incidence risk of pancreatic cancer

#### Aspirin

**Figure 2:**
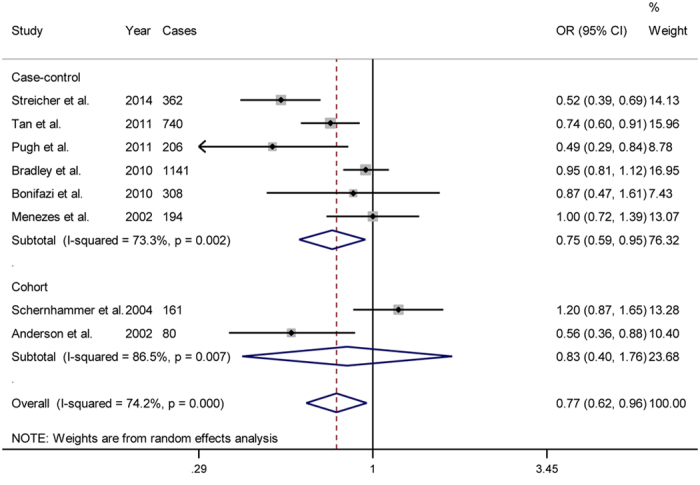
Forest plot showing the association between aspirin use and the incidence of pancreatic cancer.

Six case-control and two cohort studies were included in this combined analysis, and a pooled estimate (OR = 0.77; 95% CI = 0.62 to 0.96) revealed a decrease in the incidence of pancreatic cancer, with moderate heterogeneity amongst studies (*P* = 0.001; *I*^2^ = 74.2%; Fig. 2). The summary OR was 0.75 (95% CI = 0.59 to 0.95; *I*^2^ = 73.30%) for the case-control studies and 0.83 (95% CI = 0.40 to 1.76; *I*^2^ = 86.50%) for the cohort studies. We conducted a subgroup analyses that considered numerous covariates that might have contributed to the overall heterogeneity (for the results, see Table 2). We pre-specified five subgroups based on geographic region, gender, study quality, pattern of aspirin use, and adjustment for confounds. Notably, most of the studies were adjusted for major confounds such as age, sex, smoking status, history of diabetes and body mass index (BMI). However, not all of the included studies adjusted for important confounds such as alcohol consumption and a family history of pancreatic cancer. Therefore, we pre-specified three strata to explore whether the differences in confounds influenced the results. Significant inverse associations between aspirin use and the incidence of pancreatic cancer were observed across almost all of the strata included in the subgroup analyses. However, considerable heterogeneity was observed amongst the studies within each stratum. A sensitivity analysis revealed that the exclusion of any one study did not substantially alter the overall estimate, with an OR range of 0.72 (95% CI = 0.58 to 0.90) to 0.83 (95% CI = 0.68 to 1.02). No evidence of publication bias was found according to an Egger linear regression test (*P* = 0.413).

Furthermore, we examined the dose, frequency and duration risks of aspirin use; these data are presented in Table 3 and represent a combination of case-control and cohort studies because of the small number of studies within each stratum. Neither high-dose (OR = 0.98; 95% CI = 0.96 to 1.00; *P* = 0.097; *I*^2^ = 0.0%) nor low-dose aspirin use was significantly related to pancreatic cancer prevention (OR = 1.01; 95% CI = 0.82 to 1.24; *I*^2^ = 64.4%). Significant declines in the incidence of pancreatic cancer were related to high-frequency (OR = 0.57; 95% CI = 0.39 to 0.83; *I*^2^ = 26.1%) and medium-frequency (OR = 0.57; 95% CI = 0.38 to 0.84; *I*^2^ = 0.0%) aspirin use. Only one study^21^ reported low-frequency aspirin use, and its test statistic was not significant (OR = 0.75; 95% CI = 0.45 to 1.25). However, the summary OR associated with the analysis of each aspirin duration risk category was also non-significant (Table 2).

#### Non-aspirin NSAIDs and overall NSAIDs

**Figure 3:**
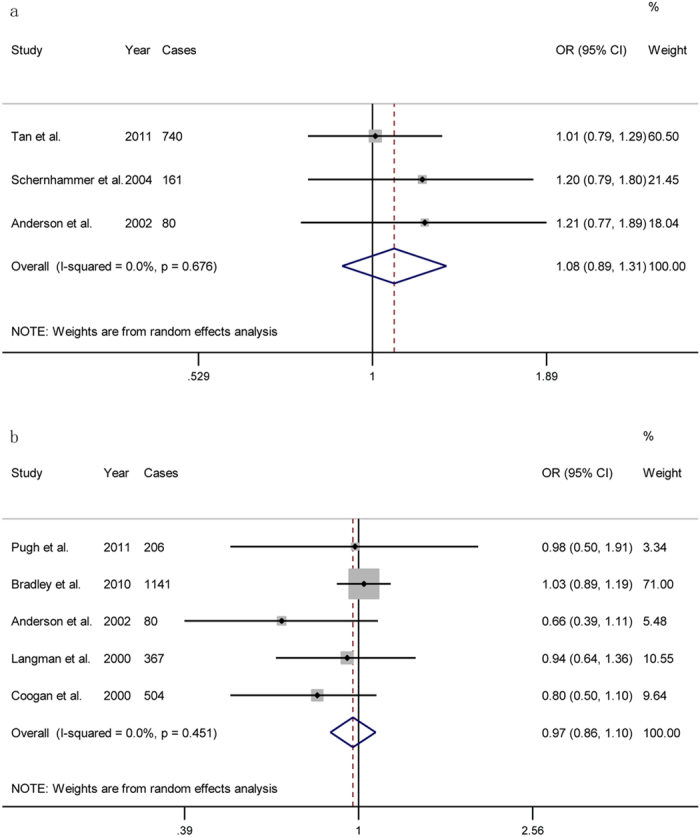
Forest plot showing the association between other NSAIDs use and the incidence of pancreatic cancer. (**a**): non-aspirin NSAIDs use; (**b**): all NSAIDs use.

The analysis of 3 (two cohorts and one case-control) studies suggested that non-aspirin NSAIDs use was not associated with a decrease in the incidence of pancreatic cancer (OR = 1.08; 95% CI = 0.90 to 1.31); these studies showed no heterogeneity (*P* = 0.676; *I*^2^ = 0.0%; Fig. 3a). In addition, 5 studies (four case-controls and one cohorts) revealed that overall NSAIDs use was not associated with a decrease in the incidence of pancreatic cancer (OR = 0.97; 95% CI = 0.86 to 1.10); these studies showed no heterogeneity (*P* = 0.451; *I*^2^ = 0.0%; Fig. 3b).

### Cancer mortality risk

**Figure 4:**
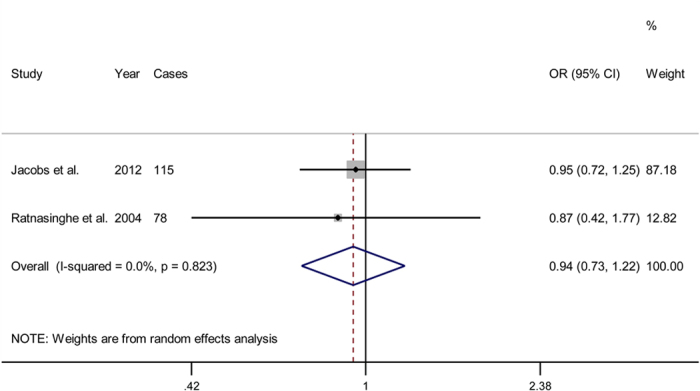
Forest plot showing the association between aspirin use and pancreatic cancer mortality.

Two cohort studies investigated the association between aspirin use and cancer-specific mortality. Together, these studies revealed that aspirin use was not associated with a decreased mortality risk (OR = 0.94; 95% CI = 0.73 to 1.22). No significant heterogeneity was observed between the 2 studies (*P* = 0.823; *I*^2^ = 0.0%; Fig. 4).

## Discussion

The current meta-analysis included approximately 258,000 participants from 12 observational studies and investigated the association between NSAID use with the rates of pancreatic cancer and mortality in real-world practice. The major findings of this meta-analysis support the mechanistic hypothesis that aspirin use (specifically, high- and medium-frequency use) is inversely related to the risk of pancreatic cancer. In contrast, neither overall NSAIDs use nor non-aspirin NSAIDs use was associated with a reduced risk of pancreatic cancer.

The main finding of our meta-analysis contradicts a previous meta-analysis (Larsson *et al*.^29^) that evaluated the effect of aspirin on pancreatic cancer risk. This previous meta-analysis included 8 studies (one case-control, one RCT and six cohorts) in its analysis of aspirin use; furthermore, it considered incidence and mortality together to represent the overall risk of pancreatic cancer. That analysis revealed that aspirin use is not associated with a reduced risk of pancreatic cancer. However, this evidence was limited because of a methodological weakness. The effect of aspirin on cancer prevention (i.e., a chemoprevention effect) might be masked by its potential therapeutic effect. We only considered observational studies in our meta-analysis to explore the effects of NSAIDs use on the incidence of pancreatic cancer in the real world because they reflect actual practice patterns better than RCTs^32^, which tend to overstate the effect of a new treatment when it is introduced to an entire target population^32^. Unlike a previous meta-analysis, however, we did not include a particular study that provided data as SIRs^13^ without adjustments. Moreover, we excluded Schreinemachers’s study^15^ from our analysis because its cohort, which is part of the first National Health and Nutrition Examination Survey (NHANES I) study, was included in a duplicate publication by Ratnasinghe *et al*.^16^. We believe that both articles were incorrectly included in the meta-analysis by Larsson *et al*.^29^. Thus, our meta-analysis, which has a larger sample size, adds to previous findings by demonstrating that non-aspirin NSAIDs and overall NSAIDs use are not associated with the incidence of pancreatic cancer.

We assessed the relationship between aspirin use and pancreatic cancer mortality and did not observe a significant association. However, this finding should be interpreted with caution because of the small number of studies (n = 2) included. In fact, these patients might have died from causes other than pancreatic cancer, thereby creating bias and obscuring the true incidence of pancreatic cancer. Unfortunately, we did not obtain the data needed to evaluate the effect of duration risk on mortality. However, an individual patient data analysis of 8 RCTs suggested that daily aspirin use over at least 5 years significantly reduces mortality due to several common cancers, including pancreatic cancer (hazard ratio (HR) = 0.25; 95% CIs = 0.07 to 0.92)^33^.

We also investigated the potential implications of aspirin use dose, frequency and duration. A meta-analysis by Cui *et al*.^31^ suggested that high-dose aspirin use (OR = 0.88; 95% CI = 0.76 to 1.01; P = 0.069), but not low-dose aspirin use (OR = 0.99; 95% CI = 0.91 to 1.07), reduces the risk of pancreatic cancer; however, the summary OR of the high-dose aspirin group was not significant. Moreover, the incidence and mortality rates associated with pancreatic cancer were pooled together to represent overall risk. Notably, when a study that investigated mortality risk was excluded^17^ from this meta-analysis, the overall risk estimates associated with the effect of high-dose aspirin use on cancer risk were significant (OR = 0.78; 95% CI = 0.64 to 0.95; *P* = 0.014)^31^. We also excluded 3 studies^16^,^18^,^21^ that were incorrectly included in the previous meta-analysis because they did not provide information on high-dose use. Using incidence as an independent endpoint, our analysis did not find a beneficial effect of high-dose aspirin use on the incidence of pancreatic cancer (OR = 0.98; 95% CI = 0.96 to 1.00; *P* = 0.097; *I*^2^ = 0.0%). In addition, we did not include 5 of the previously included studies in our analysis of the effect of low-dose aspirin use on the incidence of cancer. Of these 5 studies, one did not meet our inclusion criteria^13^, one investigated mortality risk^17^, one used a RCT design^34^, and the other 2 did not provide data regarding low-dose aspirin use^21^,^25^. Even after excluding these studies, we did not find an association between low-dose aspirin use and the incidence of pancreatic cancer. Furthermore, another meta-analysis by Capurso *et al*.^30^ did not identify chemopreventive effects associated with the low use, intermediate use, or high use of NSAID based on a combination of dose and duration. However, these authors combined aspirin and NSAIDs use in each exposure category; this strategy might have masked the effect of aspirin use alone. In addition, duration of use was not assessed separately from dose in this meta-analysis. Our meta-analysis, however, considered aspirin use individually and found that high- and medium-frequent use led to a significant decrease in the incidence of pancreatic cancer. We did not find a significant duration risk of aspirin use on the incidence of pancreatic cancer. Thus, our meta-analysis provides a clearer and more comprehensive understanding of the potential benefits of NSAID use because of its strict inclusion criteria and accumulation of evidence; moreover, it has higher statistical power than previous meta-analyses.

Our analysis identified a significant negative relationship between aspirin use and the incidence of pancreatic cancer when the case-control studies were pooled together; however, this finding was not true for the two cohort studies. However, the exposure assessments of these cohort studies were based on self-reports. Moreover, these studies were both based on women, whereas the case-control samples included both men and women. Therefore, their results might be biased, and the limited data from the 2 cohort studies might limit the interpretation. In addition, our stratified analysis based on geographic region did not reveal significant results in America or Europe, which raises interesting questions. In fact, all of the included American studies reported baseline aspirin use values exceeding 40%, which might have reduced the chance of observing small relative differences in aspirin use as a risk factor^30^. Moreover, because of the limited number of studies included in the stratified analysis of European studies, the results might be biased. Thus, additional investigations are needed.

Several mechanisms might underpin the associations observed in our study. Our meta-analysis did not find that the negative association between aspirin use and the incidence of pancreatic cancer depended on dosage. Individuals consuming high doses of aspirin primarily did so for pain control or anti-inflammation purposes^35^; we hypothesise that these reasons are associated with other serious symptoms such as the abdominal discomfort that radiates to the back that occurs during the early stages of pancreatic cancer^36^. This factor might have partially influenced the observed association. In contrast, low-dose aspirin use is commonly used for the primary or secondary prevention of cardiovascular disease^37^, and lipid-lowering drugs are often consumed in combination with aspirin to prevent coronary artery disease. This reason might have masked the effect of the reduction in the rate of pancreatic cancer mortality^38^ and explain the lack of the chemopreventive effect typically associated with low-dose aspirin use. Aspirin use over fewer than 10 years since the onset of cancer might slow carcinogenesis rather than prevent initial tumour development because of the average 10-year latency of this disease^39^. In addition, one RCT by Cook *et al*.^34^ investigated this issue and suggested that low doses of aspirin (100 mg) over a 10-year treatment did not reduce the risk of pancreatic cancer, partially supporting our findings. Our meta-analysis did not identify an association between non-aspirin NSAID use and the incidence of pancreatic cancer, perhaps because only aspirin irreversibly inactivates COX-2 enzymes^40^.

Given the poor prognosis of pancreatic cancer and the widespread use of aspirin in the general population, successful prevention might have a significant public health effect. Our meta-analysis addresses the beneficial effects of aspirin use; however, physicians should be aware of the optimal dosage and frequency of aspirin use as well as its side effects.

Several limitations should be acknowledged. First, significant heterogeneity was observed across the studies included in the current analysis. Although we stratified the data into subgroups based on geographic region, gender, study quality, pattern of aspirin use, and adjusted confounds, considerable heterogeneity remained. This result is not surprising given the discrepancies of each study with regard to their designs; the differing race, age and lifestyle factors of their participants; sample sizes; definitions of drug exposure; follow-up evaluation lengths; and, most importantly, the type and dose of aspirin used as well as its exact administration schedule.

Second, most studies adjust for major confounds such as age, sex, BMI, smoking status, and diabetes history using multivariate statistical models. However, the adjusted confounds differed across the studies, which might have affected the overall results. In addition, few studies have adjusted for a family history of pancreatic cancer, which is a significant risk factor for the disease^41^. Researchers do not always make the same decisions concerning confounds. To explore whether the differences across the adjustments for confounds influenced our results, we performed stratified analyses according to the adjustments for BMI, alcohol consumption, and pancreatic cancer history. We found that these variables did not influence the overall results.

A third limitation was the potential misclassification of aspirin use. The consumption levels of the low- and high-dose categories as well as the frequency categories varied across studies; we attempted to minimize this imprecision by pooling the most similar data across the analyses. Nevertheless, this imprecision might have created study heterogeneity in the relevant pooled analysis. Therefore, this result should be considered with caution.

Another limitation is that the quantity of studies included in the analysis was not sufficient to evaluate publication bias, although the P-value associated with the Egger’s test was non-significant (P = 0.413). Moreover, we did not find unpublished data to avoid publication bias.

Finally, our findings concerning the risks associated with the dose, frequency and duration of aspirin use should be interpreted with caution because few studies were included in the stratified analyses, and most studies lacked information regarding these variables. Of the studies that provided this information, these parameters varied across each trial and therefore would have resulted in invalid statistical analyses for these groups.

In summary, the results from the current meta-analysis suggest that aspirin use reduces the incidence of pancreatic cancer but not cancer-specific mortality in the real world. However, these findings should be interpreted with caution because of the considerable heterogeneity amongst the included studies regarding incidence risk and the limited number of included studies concerning mortality risk. Specifically, the negative association between aspirin use and the incidence of pancreatic cancer likely depends on the frequency of this use (i.e., high- and medium- vs. low-frequency doses). Moreover, our meta-analysis did not find any associations between the incidence of pancreatic cancer and either non-aspirin NSAIDs or overall NSAIDs use; the studies included in this analysis had no heterogeneity. However, the small number of studies included in the analyses might limit our interpretations. This study might provide insights that enable a better understanding of the relationship between pancreatic cancer risk and aspirin/NSAIDs use. Future research, especially prospective studies, should be conducted to validate our findings and investigate whether the relationship between aspirin use and the incidence of pancreatic cancer depends on dose or duration of use.

## Methods

This study was conducted using the Cochrane methodology and was reported in accordance with the Preferred Reporting Items for Systematic Reviews and Meta-Analyses (PRISMA) guidelines^42^ (Supplementary Table S2).

### Search strategy

We searched PubMed and Embase for studies of pancreatic cancer risk and aspirin/other NSAID use published in any language from the inception dates of these databases to June 2015. We searched ClinicalTrials.gov for unpublished studies. The search terms included the generic names of individual drugs, their therapeutic classes, and pancreatic cancer outcome terms (Supplementary Table S3). No restrictions were applied. Furthermore, we reviewed the reference lists of the retrieved articles and recent reviews to identify additional potentially relevant studies.

### Study selection

Studies were included when they met the following criteria: 1) a cohort or case-control design was used; 2) the exposure of interest was any NSAID use, including aspirin, non-aspirin NSAIDs and overall NSAIDs; 3) the incidence rate, mortality rate, or both of pancreatic cancer was assessed; and 4) an adjusted OR or relative risk (RR) with 95% CIs were provided. Regarding duplicate publications^15^,^16^,^17^,^23^, only the most informative and complete studies were included^16^,^23^.

### Data extraction

The following information was abstracted from all of the included studies using a standardized data collection form: study name (together with the first author’s name and year of publication), study design, study period, study follow-up, study sample size (including both the numbers of cases and controls or the cohort size), study outcomes, the quality score of each study, the types of NSAIDs and consumption categories employed, the ORs and RRs with corresponding 95% CIs for each category, and the adjusted confounds. We also reviewed the supplementary files of each study and contacted the authors for more detailed information when necessary.

### Assessments of quality and risk of bias across studies

We assessed the authenticity and quality of the included studies using the NOS^43^. The NOS evaluates a study from 3 broad perspectives and awards a maximum score of 9 points. We assigned the following risk of bias categories based on the NOS score of each study: low risk of bias (7–9 points), medium risk of bias (4–6 points), and high risk of bias (less than 4 points).

Two investigators (YP Zhang and YD Wan) independently conducted the literature search, study selection, and data extraction as well as the assessments of quality and risk of bias across studies. Any discrepancies were resolved via consensus.

### Statistical analyses

The measure of an effect was its associated OR and 95% CIs. Because the absolute risk of pancreatic cancer is low, we generally ignored the distinctions amongst the various measures of relative risk (ORs in case-control studies and RRs in cohort studies); therefore, we reported all of the results as ORs for simplicity^44^. Multivariate adjusted ORs were pooled in the analysis to minimise potential confounding bias. Between-study heterogeneity was assessed using the Cochran Q statistic (significance level at *P* < 0.1) and by estimating *I*^2^ 45. Studies with an *I^2^* statistic of 25% to 50%, 50% to 75%, and >75% were regarded as low heterogeneity, moderate heterogeneity, and high heterogeneity, respectively^45^. The DerSimonian and Laird^46^ random-effects model, which incorporates both within- and between-study variability, was used regardless of heterogeneity. The inverse-variance method was used to pool the adjusted ORs. We also conducted a sensitivity analysis using the one-study-out method to test the robustness of the pooled estimate. We intended to assess the publication bias across studies using Egger’s linear regression test^47^ at the 90% level; however, no testing for funnel plot asymmetry was conducted because of the small number of studies included in all analyses (n < 10)^48^.

To achieve a clear understanding of the relationship between NSAID use and the incidence of pancreatic cancer, we stratified the data based on drug type (aspirin, non-aspirin NSAIDs, overall NSAIDs) and included two outcomes (the incidence and mortality rates of pancreatic cancer). The effect estimates were pooled both overall and by study design in the aspirin use analysis. Overall NSAIDs use in this report refers to the studies that reported NSAIDs use but did not provide ratios for the use of aspirin or non-aspirin NSAIDs alone. To analyse the dose, frequency, and duration risks associated with aspirin use, we pooled similar data in each category to conduct the analysis. We defined low-dose aspirin use as 100 mg per day and high-dose aspirin use as 300 mg per day. Low-, medium-, and high-frequency aspirin use were defined as ≤1 day per week, 2–5 days per week, and ≥6 days per week, respectively. A two-tailed P-value of <0.05 was considered significant. All statistical analyses were performed using STATA 12.0 (StataCorp, College Station, TX).

## Additional Information

**How to cite this article**: Zhang, Y.-P. *et al*. Aspirin might reduce the incidence of pancreatic cancer: a meta-analysis of observational studies. *Sci. Rep.* **5**, 15460; doi: 10.1038/srep15460 (2015).

## Supplementary Material

Supplementary Table 1

Supplementary Table 2

Supplementary Table 3

## Figures and Tables

**Table 1:** Main characteristics included in the meta-analysis.

**First Author [Country] [Ref.]/Year**	**Design**	**Study Period**	**Follow Up (year)**	**Cases**	**Controls or Cohort Size**	**Exposure**	**Definition of NSAID Use**	**Strength of Association (95% CI)**	**Confounders For Adjustment**	**Outcome**	**Score**
Streicher *et al*.[US]/2014^22^	Case-control	2005–2009	NA	362	690	aspirin	never used	OR, 1	age, sex, race, smoking status, BMI, diabetes, blood type, education	Incidence	8^a^
							regularly used	OR, 0.52(0.39–0.69)			
							low-dose (75–325 mg per day) aspirin	OR, 0.94(0.91–0.98)			
							regular-dose (325–1200 mg every 4 to 6 hours) aspirin	OR, 0.98(0.96–1.01)			
							aspirin ≤ 6 y	OR, 0.50(0.36–0.70)			
							aspirin >10 y	OR, 0.61(0.37–1.00)			
Jacobs *et al*.[US]/2012^23^	Cohort	1992–2008	17	115	100139	aspirin	never used	RR, 1	age, sex, race, smoking status, BMI, heart disease, stroke, diabetes, hypertension, cholesterol-lowering drug use, aspirin use, NSAID use, history of colorectal endoscopy, physical activity level, education	Mortality	8^a^
							updated analyses for current daily use	RR, 0.95(0.72–1.25)			
							updated analyses for aspirin use <5 y	RR, 0.89(0.64–1.23)			
							updated analyses for aspirin use ≥5 y	RR, 1.03(0.73–1.46)			
Tan *et al*.[US]/2011^19^	Case-control	2004–2010	NA	740	1043	aspirin/non-aspirin NSAIDs	aspirin never used (<1day/month)	OR, 1	age, sex, smoking status, BMI, diabetes	Incidence	6^a^
							aspirin ever used (≥1day/month)	OR, 0.74(0.60–0.91)			
							aspirin frequency of use 2–5 days/week	OR, 0.61(0.38–0.96)			
							aspirin frequency of use 6+ days/week	OR, 0.63(0.47–0.85)			
							aspirin dosage of 1–2 tablets/day	OR, 0.81(0.63–1.03)			
							aspirin dosage of 3+ tablets/day	OR, 0.72(0.50–1.04)			
							non-aspirin NSAIDs used (≥1 day/month)	OR, 1.01(0.79–1.29)			
Pugh *et al*.[UK]/2011^20^	Case-control	2004–2007	NA	206	251	aspirin/NSAIDs	never used	OR, 1	age, sex, smoking status, diabetes	Incidence	6^a^
							aspirin use	OR, 0.49(0.29–0.84)			
							NSAIDs use	OR, 0.98(0.50–1.91)			
Bradley *et al*.[UK]/2010^25^	Case-control	1995–2006	NA	1141	7954	aspirin/NSAIDs	never used	OR, 1	smoking status, BMI, alcohol use, history of chronic pancreatitis, history of rheumatoid arthritis, use of other drugs, diabetes, prior cancer	Incidence	7^a^
							any use of an NSAID until 1 year before diagnosis	OR, 1.03(0.89–1.19)			
							duration of low-dose NSAIDs (<1.0 DDD per day) >5 y	OR, 0.70(0.49–0.99)			
							duration of high-dose NSAIDs (≥1.0 DDD per day) >5 y	OR, 0.85(0.53–1.36)			
							high-dose NSAIDs (1–200 DDDs per day)	OR, 0.99(0.94–1.03)			
							ever used for aspirin and derivatives until 1 year before diagnosis	OR, 0.95(0.81–1.12)			
							high-dose aspirin (≥300 mg a day)	OR, 1.10(0.81–1.50)			
Bonifazi *et al*.[Italy]/2010^18^	Case-control	1991–2008	NA	308	477	aspirin	non-regular used (<1 day/week for more than 6 months)	OR, 1	age, sex, smoking status, BMI, diabetes, education, study center, year of interview	Incidence	8^a^
							regular used (≥1 day/week for more than 6 months)	OR, 0.87(0.47–1.61)			
							duration of use <5 y	OR, 1.40(0.62–3.17)			
							duration of use ≥5 y	OR, 0.53(0.21–1.33)			
							current users ≥5 y	OR, 0.23(0.06–0.90)			
Schernhammer *et al*.[US]/2004^14^	Cohort	1980–1998	18	161	88378	aspirin/non-aspirin NSAIDs	non-regular used (<2 tablets per week)	RR, 1	age, smoking status, BMI, diabetes, non-vigorous physical activity in metabolic equivalents per week, follow-up cycle	Incidence	7^a^
							use of non-aspirin NSAIDs	RR, 1.20(0.79–1.80)			
							regular use (≥2 tablets per week)	RR, 1.20(0.87–1.65)			
							current aspirin use 1–3 tablets per week	RR, 1.26(0.85–1.85)			
							current aspirin use 4–6 tablets per week	RR, 1.41(0.82–2.40)			
							current aspirin use 7–13 tablets per week	RR, 1.65(1.05–2.59)			
							current aspirin use ≥14 tablets per week	RR, 0.86(0.39–1.89)			
							non-regular used (<5 tablets of aspirin per week)	RR, 1			
							regular use, 1–5 y	RR, 1.12(0.72–1.74)			
							regular use, 6–10 y	RR, 1.10(0.64–1.89)			
							regular use, >10 y	RR, 1.75(1.18–2.60)			
Ratnasinghe *et al*.[US]/2004^16^	Cohort	1971–1992	21	78	22756	aspirin	no aspirin used	RR, 1	age, sex, race, smoking status, BMI, poverty index, education	Mortality	9^a^
							any aspirin used (≥1 times a week for at least 6 months)	RR, 0.87(0.42–1.77)			
Anderson *et al*.[US]/2002^21^	Cohort	1992–1999	7	80	28283	aspirin/non-aspirin NSAIDs/NSAIDs	never used	RR, 1	age, smoking status, current multivitamin use, diabetes	Incidence	7^a^
							use of only aspirin	RR, 0.56(0.36–0.88)			
							use of NSAIDs	RR, 0.66(0.39–1.11)			
							use of non-aspirin NSAIDs	RR, 1.21(0.77–1.89)			
							≤1 time/week of aspirin	RR, 0.75(0.45–1.25)			
							2–5 times/week of aspirin	RR, 0.47(0.22–0.98)			
							≥6 times/week of aspirin	RR, 0.40(0.20–0.82)			
Menezes *et al*.[US]/2002^27^	Case-control	1982–1998	NA	194	582	aspirin	non-regular used	OR, 1	age, sex, race, smoking status, BMI, family history of pancreatic cancer, education	Incidence	6^a^
							regular used (at least once a week for six consecutive months)	OR, 1.00(0.72–1.39)			
							Dosage of 1–6 tablets/week	OR, 1.15(0.79–1.67)			
							dosage of ≥7 tablets/week	OR, 0.85(0.49–1.45)			
							duration of use for 0.5–10 years	OR, 0.82(0.54–1.26)			
							duration of use for ≥11 years	OR, 1.21(0.81–1.82)			
Langman *et al*.[UK]/2000^24^	Case-control	1993–1995	NA	367	1139	NSAIDs	no use	OR, 1	age, smoking status	Incidence	8^a^
							1 prescription for NSAIDs	OR, 0.94(0.64–1.36)			
							2–6 prescriptions for NSAIDs	OR, 1.08(0.75–1.54)			
							≥7 prescriptions for NSAIDs	OR, 1.49(1.02–2.18)			
Coogan *et al*.[US]/2000^26^	Case-control	1997–1998	NA	504	5952	NSAIDs	never used	OR, 1	age, sex, race, religion, smoking status, alcohol use, family history of digestive cancer, education, interview year, study center	Incidence	6^a^
							continuing regular NSAIDs use (initiated ≥1 y previously)	OR, 0.8(0.5–1.1)			
							duration of NSAIDs use <5 y	OR, 0.8(0.5–1.4)			
							duration of NSAIDs use ≥5 y	OR, 0.6(0.4–1.1)			

a: quality assessment by Newcastle-Ottawa Scales; b: quality assessment by jaded score; NA: not available; y: year; NSAID: non-steroidal anti-inflammatory drug; CI: confidence interval; OR: odds ratio; RR: relative risk; DDD: defined daily dose.

**Table 2:** Subgroup analysis for aspirin use on pancreatic cancer incidence.

**Study characteristics**	**Number of Studies**	**OR (95% CI)**	***Ρ ***^**a**^	***Ρ ***^**b**^	***I*****^2^**
Total	8	0.77(0.62–0.96)	0.000		74.2%
Geographic region				0.97	
America^14^,^19^,^21^,^22^,^27^	5	0.77(0.57 to 1.03)	0.001		79.1%
Europe^18^,^20^,^25^	3	0.77(0.52 to 1.16)	0.065		63.4%
Gender				0.79	
Male and female^18^,^19^,^20^,^22^,^25^,^27^	6	0.75(0.59 to 0.95)	0.002		73.3%
Female^14^,^21^	2	0.83(0.40 to 1.76)	0.007		86.5%
Study quality				0.85	
Low risk of bias^14^,^18^,^21^,^22^,^25^	5	0.79(0.57 to 1.09)	0.000		81.0%
Medium risk of bias^19^,^20^,^27^	3	0.75(0.55 to 1.03)	0.069		62.5%
Pattern of aspirin use				0.50	
Ever use^19^,^20^,^21^,^25^	4	0.72(0.55 to 0.94)	0.015		71.4%
Regularly use^14^,^18^,^22^,^27^	4	0.85(0.56 to 1.30)	0.001		82.0%
Adjustment for confounders					
BMI				0.03	
Yes^14^,^18^,^19^,^22^,^25^,^27^	6	0.85(0.67 to 1.06)	0.001		75.4%
No^20^,^21^	2	0.53(0.38 to 0.75)	0.706		0.0%
Family history of pancreatic cancer				0.15	
Yes^27^	1	1.00(0.72 to 1.39)	–		–
No^14^,^18^,^19^,^20^,^21^,^22^,^25^	7	0.74(0.58 to 0.94)	0.000		76.5%
Alcohol consumption				0.10	
Yes^25^	1	0.95(0.81 to 1.12)	–		–
No^14^,^18^,^19^,^20^,^21^,^22^,^27^	7	0.74(0.57 to 0.95)	0.001		72.6%

a: *P* value for heterogeneity within each subgroup; b: *P* value for heterogeneity between subgroups; OR: odds ratio; BMI: body mass index; CI: confidence interval.

**Table 3:** Dose-, frequency-, and duration-risk of aspirin use for pancreatic cancer incidence.

**Study characteristics**	**Number of Studies**	**Number of Cases**	**OR (95% CI)**	***Ρ***_**heterogeneity**_	***I*****^2^**
Total	8	4,256	0.77(0.62–0.96)	0.000	74.2%
Dose					
Low dose^14^,^19^,^22^,^27^	4	1,457	1.01(0.82–1.24)	0.037	64.40%
High dose^14^,^19^,^22^,^25^,^27^	5	2,926	0.98(0.96–1.00)	0.459	0.00%
Frequency					
Low frequency^21^	1	80	0.75(0.45–1.25)	–	–
Medium frequency^19^,^21^	2	820	0.57(0.38–0.84)	0.561	0.00%
High frequency^19^,^21^	2	820	0.57(0.39–0.83)	0.245	26.10%
Duration					
duration less than 5y^14^,^18^,^22^,^27^	4	1,025	0.84(0.54–1.32)	0.01	73.50%
duration more than 5y^18^	1	308	0.53(0.21–1.33)	–	–
duration more than 10y^14^,^22^,^27^	3	717	1.11(0.63–1.96)	0.005	81.10%

OR: odds ratio; y: year.
