# Supplementary material for: Aspirin might reduce the incidence of pancreatic cancer: A meta-analysis of observational studies
Source: Sci Rep. 2015 Oct 21;5:15460. doi: 10.1038/srep15460 (PMC4614261; doi:10.1038/srep15460)
Supplement: Supplementary Table 1 [file srep15460-s1.pdf]

Aspirin might reduce the incidence of pancreatic cancer: a meta-analysis of observational studies

Yan-Peng Zhang[1], You-Dong Wan[2], Yu-Ling Sun[1]*, Jian Li[1], Rong-Tao Zhu[1]

Affiliations:

[1]Institute of Hepatobiliary and Pancreatic Diseases, Zhengzhou University, Department of Hepatobiliary and Pancreatic Surgery, The First Affiliated Hospital of Zhengzhou University, School of Medicine, Zhengzhou, P. R. China;

[2]Department of Integrated Intensive Care Unit, the First Affiliated Hospital, Zhengzhou University, Zhengzhou, China;

*__Correspondence:__ Dr. Yu-Ling Sun, Institute of Hepatobiliary and Pancreatic Diseases, Zhengzhou University, Department of Hepatobiliary and Pancreatic Surgery, The First Affiliated Hospital of Zhengzhou University, School of Medicine, 1 Jianshe Road, Zhengzhou 450052, P.R. China; Telephone: +86 037167967126, Email: ylsun@zzu.edu.cn.

Supplementary Table S1. Methodological quality assessment (risk of bias) of included studies by Newcastle-Ottawa Scales.

**Study**	**Selection**				**Comparability**	**Outcome**			**Total score**
	Exposed Cohort / Case Definition	Nonexposed Cohort / Case Representativeness	Ascertainment of Exposure / Selection of Controls	Outcome of Interest / Definition of Controls		Assessment of Outcome	Length of Follow-up / Same Ascertainment	Adequacy of Follow-up / Non-Response rate	
Streicher et al. /2014	*	*	*	*	**	*	*	-	8
Jacobs et al. /2012	*	*	*	*	*	*	*	*	8
Tan et al. /2011	*	*	-	*	*	*	*	-	6
Pugh et al. /2011	*	*	-	*	*	*	*	-	6
Bradley et al. /2010	*	*	*	-	**	*	*	-	7
Bonifazi et al. /2010	*	*	-	*	**	*	*	*	8
Schernhammer et al. /2004	*	*	*	*	*	*	*	-	7
Ratnasinghe et al. /2004	*	*	*	*	**	*	*	*	9
Anderson et al. /2002	-	*	*	*	**	*	*	-	7
Menezes et al. /2002	*	*	-	*	*	*	*	-	6
Langman et al. /2000	*	*	-	*	**	*	*	*	8
Coogan et al. /2000	*	*	-	*	*	*	*	-	6
