# Supplementary material for: Aspirin might reduce the incidence of pancreatic cancer: A meta-analysis of observational studies
Source: Sci Rep. 2015 Oct 21;5:15460. doi: 10.1038/srep15460 (PMC4614261; doi:10.1038/srep15460)
Supplement: Supplementary Table 3 [file srep15460-s3.pdf]

Aspirin may reduce the incidence of pancreatic cancer: a meta-analysis of observational studies

Yan-Peng Zhang[1], You-Dong Wan[2], Yu-Ling Sun[1]*, Jian Li[1], Rong-Tao Zhu[1]

Affiliations:

[1]Institute of Hepatobiliary and Pancreatic Diseases, Zhengzhou University, Department of Hepatobiliary and Pancreatic Surgery, The First Affiliated Hospital of Zhengzhou University, School of Medicine, Zhengzhou, P. R. China;

[2]Department of Integrated Intensive Care Unit, the First Affiliated Hospital, Zhengzhou University, Zhengzhou, China;

***Correspondence:** Dr. Yu-Ling Sun, Institute of Hepatobiliary and Pancreatic Diseases, Zhengzhou University, Department of Hepatobiliary and Pancreatic Surgery, The First Affiliated Hospital of Zhengzhou University, School of Medicine, 1 Jianshe Road, Zhengzhou 450052, P.R. China; Telephone: +86 037167967126, Email: ylsun@zzu.edu.cn.

Supplementary Table S3. Review methodology of the meta-analysis.

**Databases**	PubMed, Embase	**No. references retrieved after duplication**
**Search terms #1**	(pancrea* cancer) OR (pancrea* carcinoma) OR (pancrea* neoplasms)	101,787
**Search terms #2**	(anti-inflammatory agents) OR (non-steroidal anti-inflammatory drugs) OR (NSAID*) OR (selective non-steroidal anti-inflammatory drugs) OR (nonselective non-steroidal anti-inflammatory drugs) OR (cyclo-oxygenase-2 inhibitors) OR (cox-2 inhibitors) OR (selective cyclo-oxygenase-2 inhibitors) OR (selective cox-2 inhibitors) OR (aspirin) OR (acetylsalicylic acid) OR (celecoxib) OR (rofecoxib) OR (meloxicam) OR (lumiracoxib) OR (valdecoxib) OR (parecoxib) OR (etoricoxib) OR (diclofenac) OR (ibuprofen) OR (naproxen) OR (piroxicam) OR (indomethacin) OR (indomethacin) OR (azapropazone) OR (etodolac) OR (fenbufen) OR (fenoprofen) OR (flurbiprofen) OR (ketoprofen) OR (ketorolac) OR (mefenamic acid) OR (nabumetone) OR (sulindac) OR (tenoxicam) OR (tiaprofenic acid)	509,707
**Search terms #3**	Search terms #1 AND search terms #2	2,435
